# Educational Attainment and Prevalence of Cardiovascular Health (Life’s Simple 7) in Asian Americans

**DOI:** 10.3390/ijerph18041480

**Published:** 2021-02-04

**Authors:** Md Towfiqul Alam, Sandra E. Echeverria, Melissa J. DuPont-Reyes, Elizabeth Vasquez, Rosenda Murillo, Tailisha Gonzalez, Fatima Rodriguez

**Affiliations:** 1 Department of Public Health Education, University of North Carolina at Greensboro, Greensboro, NC 27412, USA; seecheve@uncg.edu; 2 Department of Epidemiology & Biostatistics, Texas A&M University, College Station, TX 77843, USA; melissadr@exchange.tamu.edu; 3 Department of Epidemiology & Biostatistics, University at Albany, Albany, NY 12144, USA; evasquez2@albany.edu; 4 Department of Psychological, Health, & Learning Sciences, University of Houston, Houston, TX 77204, USA; rmurill3@central.uh.edu; 5 Department of Community Health, CUNY Graduate School of Public Health & Health Policy, New York, NY 10027, USA; Tailisha.Gonzalez59@sphmail.cuny.edu; 6 Division of Cardiovascular Medicine, Stanford University, Quarry Road, Falk CVRC, Stanford, CA 94305, USA; frodrigu@stanford.edu

**Keywords:** asian american, education, nativity, length of stay, cardiovascular health

## Abstract

Asian Americans have a high burden of cardiovascular disease, yet little is known about the social patterning of cardiovascular health (CVH) in this population. We examined if education (<high school diploma, high school diploma, some college, and college degree+) was associated with CVH and if this varied by time in the United States (U.S.). Our study population included Asian Americans 20+ years of age sampled in the 2011-16 National Health and Nutrition Examination Survey (n = 1634). Ideal cardiovascular health was based on a composite score of adiposity, total cholesterol, blood pressure, blood glucose, smoking, physical activity, and diet. We fit sequential weighted multivariate logistic regression models for all analyses. The prevalence of ideal cardiovascular (CV) health was 17.1% among those living in the U.S. <10 years, 7.1% for those living in the U.S. >10+ years, and 15.9% for the U.S.-born. All models showed that low education compared to high education was associated with lower odds of having ideal CVH. This pattern remained in adjusted models but became non-significant when controlling for nativity (odds ratio = 0.34, 95% confidence interval: 0.10, 1.13). Models stratified by time in the U.S. were less consistent but showed similar education gradients in CVH. Low education is a risk factor for attaining ideal cardiovascular health among Asian Americans, regardless of time in the U.S.

## 1. Introduction

Cardiovascular disease is the leading cause of mortality and morbidity in the United States (U.S.). A total of 655,000 Americans die from heart disease each year, representing one of four of all national deaths [1,2,3]. According to the Centers for Disease Control and Prevention (CDC), an American experiences a myocardial infarction every 40 s [4]. While the mortality rate associated with cardiovascular disease has decreased over the years, health improvement is not equitably shared across racial and ethnic subgroups [4]. Research shows Asian Americans have higher mortality related to cardiovascular disease compared to Non-Hispanic Whites [5,6]. The largest Asian American groups in the U.S. include people of Chinese, Filipino, Indian, Vietnamese, Korean, and Japanese origin and, in recent years, have increasingly represented people with varying levels of socioeconomic position, including educational backgrounds [7]. Moreover, Asian Americans are one of the fastest-growing racial/ethnic groups in the U.S., currently representing 22.2 million individuals and projected to reach 37 million by 2060 [8,9]. Thus, research is urgently needed to identify potential causes of cardiovascular disparities in Asian Americans as the population grows and ages over the next few decades.

In 2010, the American Heart Association (AHA) provided new guidelines for assessing and determining success in reducing cardiovascular disease. Specifically, AHA proposed to shift measures of disease to one of cardiovascular health (CVH) by examining seven cardiovascular risk factors or health behaviors, known as Life’s Simple 7 (LS7). These measures include an assessment of adiposity, total cholesterol, blood pressure, blood glucose, smoking status, physical activity, and diet [10,11,12]. The guideline and metrics are a pragmatic tool for policy and clinical recommendations related to cardiovascular health. In addition, AHA stressed the importance of monitoring racial/ethnic disparities in the application of these new metrics in order to reach its goal of improving CVH by 20% in the year 2020 AHA goals [13]. In addition to the Life’s Simple 7 Goal, the American Heart Association recently released the 2030 impact goal, further supporting the need for continued surveillance of cardiovascular health metrics across population groups [14].

Despite more than ten years since the release of the Life’s Simple 7 framework, little work exists examining the role of social determinants in shaping cardiovascular health, particularly for Asian Americans. Prevention or management of CVH factors are rooted in determinants, such as income, a neighborhood of residence, access to health insurance, and educational attainment [15]. Although a vast literature exists documenting social determinants of cardiovascular disease [16,17,18,19,20,21], little research exists examining educational attainment as a social determinant of Asian American health. Moreover, other research has pointed out that Asian Americans are often seen as a socially advantaged group with less effort needed to address health disparities in this group [22]. Our study advanced the literature in two ways. First, we tested for social gradients in cardiovascular (CV) health, given stereotypes of Asian Americans as all being highly educated and thus not experiencing health disparities. Second, some evidence in the U.S. has shown a ‘flattened’ social gradient in health for immigrant groups, likely due to the generally better health of the foreign-born. The extent to which social gradients in cardiovascular health are present in Asian Americans is unclear [23,24]. Moreover, although previous studies have examined select CVH metrics among Asian Americans or used the Life’s Simple 7 CVH metrics, no study of which we are aware has considered the intersection of education and length of stay in the U.S. in shaping cardiovascular health in Asian Americans [7,25]. Length of stay or duration in the U.S. can increase cardiovascular risk due to increased exposure to obesogenic environments, consumption of unhealthy diets, less physical activity, and acculturative-stress processes [26,27,28].

In the present study, we combined six years of data from the National Health and Nutrition Examination Survey (NHANES) to (1) examine educational gradients in the prevalence of CVH in Asian Americans following the Life’s Simple 7 framework and (2) test whether the education-CVH relation varies by length of stay in the U.S. We hypothesized that Asian Americans with lower education would have poor cardiovascular health, with worse profiles found among immigrants with longer duration in the U.S. Examining the association between educational attainment and cardiovascular health in Asian American populations is imperative, given the growing size of this population and their diverse ethnic and socioeconomic background.

## 2. Methods

*Data source and study population:* NHANES is a nationally representative cross-sectional sample of the non-institutionalized U.S. population designed to assess the health and nutritional status of adults and children in the U.S. All data and materials have been made publicly available at the National Center for Health Statistics (NCHS) website [29]. NHANES uses self-reported and laboratory assessments of a range of health conditions [30]. Participants complete an in-home questionnaire and attend a mobile examination center (MEC) to collect high-quality data in a standardized environment. From 2011–2012, NHANES began oversampling Asian American populations in addition to other racial/ethnic groups. We combined NHANES data for 2011–2016 to ensure adequate estimates of the target population and our study question. Additionally, we merged NHANES data (2011–2016) with the Food Pattern Equivalent Database (FPED) for 2011 to 2016 to calculate the Healthy Eating Index-2015 (HEI-2015) component and total diet scores [31]. The Food Patterns Equivalents Database (FPED) converts the foods and beverages consumed by participants over a 2-day period to equivalent amounts outlined in 37 United States Department of Agriculture (USDA) Food Patterns components. Specifically, food patterns are measured as cup equivalents of fruit, vegetables, and dairy; ounce equivalents of grains and protein foods; teaspoon equivalents of added sugars; gram equivalents of solid fats and oils; the number of alcoholic drinks. Information on the amounts of these dietary constituents, such as fruit, vegetables, oils, added sugars, enables the calculation of component and total Healthy Eating Index-2015 (HEI-2015) scores (see Data Analysis) [31,32].

The 3 combined NHANES cycles from 2011 to 2016 had a total of 1634 Asian Americans who were aged ≥20 years and were not pregnant. In NHANES, Asians are defined as people with origin in the Far East, Southeast Asia, or the Indian subcontinent, including Cambodia, China, India, Japan, Korea, Malaysia, Pakistan, the Philippine Islands, Thailand, or Vietnam [33,34]. However, to protect the confidentiality of study participants, ethnic group data are not released, given small numbers for some ethnic groups. Among the 1634 participants who identified as Asian American, 226 were U.S.-born, and 1397 were foreign-born (data on 11 participants were missing). Participants were excluded from the primary sample if they were from races other than Asian American, <20 years of age, and pregnant (total n = 22,284), were missing information on demographic characteristics (n = 104), and were missing any information on the CVH metrics, including body mass index (BMI) (n = 11), dietary intake (n = 20), blood cholesterol (n = 89), blood glucose (n = 68), and blood pressure (n = 50). The final analytic dataset included participants who self-identified as Asian American, were not pregnant at the time of the interview, and were 20 years of age or older.

*Dependent variable:* Cardiovascular health is based on present recommendations of major risk factors and behaviors recommended by AHA. The measures include adiposity, total cholesterol, blood pressure, blood glucose, smoking status, physical activity, and diet [12]. Body mass index (BMI) was obtained using directly measured height and weight (kg/m^2^), and an Asian-specific cut-off of BMI ≥ 25 kg/m^2^ was applied to define obesity [12]. There are varying cutoffs used to define obesity among Asian Americans, given their higher risk for cardiovascular risk at lower BMI thresholds. We used a mid-point value in our study. For example, the WHO stipulates that a range of anywhere between 25 and 27 is appropriate [35]. The American Diabetes Association recommends screening for diabetes with a BMI as low as 23 [36]. Therefore, a cut off of 25 falls in line with current recommendations and follows prior research [7,37,38,39,40]. Blood samples were collected at the MEC and used to assess cholesterol. After collection of samples, they were processed, stored, and shipped to the University of Minnesota, Minneapolis, MN, for analysis. If participants were appointed to a morning session, they were asked to fast for 9 h. Participant’s fasting status was assessed by the MEC phlebotomist prior to the blood draw [16,25,41]. Ideal cholesterol level was based on a total cholesterol reading <200 mg/dL and not being treated with lipid-lowering medication, intermediate level as total cholesterol between 200 and 239 mg/dL or treated to goal, and poor status as total cholesterol of ≥240 mg/dL [12,42]. Blood pressure was measured using standardized protocols. After 5 min of quiet rest in a seated position and after determining the participant’s highest inflation level, three consecutive blood pressure (BP) readings were obtained. The average measurement of the three was used. If a BP measurement was interrupted or incomplete, a fourth attempt was made. All BP measurements (systolic and diastolic) were recorded in the MEC [16,43]. Blood pressure status was defined as ideal if the systolic blood pressure (SBP) was <120 mm Hg and diastolic blood pressure (DBP) <80 mm Hg and the participant was not using any antihypertensive medications. Intermediate status was defined as SBP = 120–129 mm Hg or DBP < 80 mm Hg or treated to goal, and poor status was classified as SBP ≥ 130 mm Hg or DBP ≥ 80 mm Hg [12,44]. NHANES only collects fasting plasma glucose (FPG) on a subset of participants, and hence, we classified diabetes risk using glucose levels obtained from hemoglobin A1C (HbA1c) tests. Ideal glucose level included those with HbA1C < 5.7% and not taking diabetes medications, intermediate status was defined as those with HbA1C= 5.7–6.4% or treated to goal, and poor status was defined as HbA1C ≥ 6.5% [12,42]. In this study, smoking was ‘ideal’ status if the respondent never smoked, had used fewer than 100 cigarettes in a lifetime, or was a former smoker who quit >1 year ago. Intermediate smoking status included those who smoked ≥100 cigarettes but who quit <1 year ago. Poor smoking status included those who smoked ≥100 cigarettes in life and was smoking every day or somedays [45]. Participants completed the internationally validated physical activity questionnaire (PAQ), which assesses frequency and duration of activities over the past week or month, including moderate (causes small increases in breathing or heart rate) and vigorous-intensity (causes large increases in breathing or heart rate) activities [46]. Ideal physical activity (PA) was defined as ≥150 min of moderate-intensity activities per week or ≥75 min of vigorous-intensity activities per week, or an equivalent combination of both. Intermediate PA was defined as 1–149 min of moderate-intensity activities per week or 1–74 min of vigorous-intensity activities per week. Poor status of PA was defined as doing no PA [47,48]. Ideal diet intake was based on the Healthy Eating Index (HEI-2015), which is the measure of diet quality based on the 2015–2020 Dietary Guidelines for Americans. It is the same as in the HEI-2010, except saturated fat and added sugars replace empty calories, with the result being 13 components. Among the 13 components, 9 support diet adequacy (total fruit, whole fruit, total vegetables, greens and beans, whole grains, dairy, total protein foods, seafood and plant proteins, and fatty acids), and 4 should be consumed in moderation (refined grains, sodium, added sugar, and saturated fats) [49]. The HEI-2015 total score was derived from the summation of the individual component scores. A total score <51 was categorized as a poor diet, 51 to 80 indicated a moderately healthy diet, and ≥81 was an ideal diet [25]. CVH metrics categorized into ideal, intermediate, and poor categories. CVH metrics score ranged from 0 to 7 by recoding each metric of CVH as a dichotomous variable in which 1 point was assigned for ideal status and 0 points for intermediate and poor status. We then created a global Life’s Simple 7 binary score where participants with a total score of 6 or more on all CVH metrics were classified as having ideal CVH, and participants with a total score of 0–5 on all CVH metrics were classified as having non-ideal CVH [16].

*Independent variables:* Our first set of analyses documented educational gradients in ideal CVH individuals who self-identified as Asian American. Educational attainment was classified as having less than a high school diploma, high school diploma, some college, and college degree or more. The number of years living in the U.S. served as a proxy for acculturation-related processes, which might influence our main association of education and cardiovascular health [50]. Given the age distribution of Asian Americans, for effect measure modification models, Asians were categorized as living in the U.S. for less than 10 years and 10 or more years. Additional covariates included sex assigned at birth (male, female), income (<$25,000, $25,000–$74,999, and ≥$75,000), age (20–39 years, 40–65 years, >65 years), and nativity status (born outside the U.S., born in the U.S.).

*Statistical analysis:* We merged 6 years of data and created variables that incorporated stratum and sampling weights in order to accommodate sampling design changes between 2011 and 2016. First, we generated analytic sample descriptive statistics for the variables of interest. Data on variables were expressed as means and standard errors for continuous variables or as percentages (based on weighted frequency) and frequency for categorical variables and presented by nativity status and length of stay in the U.S. We used χ^2^ tests to compare differences in baseline variables according to the three subgroups (U.S.-born, foreign-born living in the U.S. ≥10 years, foreign-born living in the U.S. <10 years) of Asians. We used ANOVA tests to compare differences in baseline mean age for the same three subgroups. We used χ^2^ tests to compare differences in the prevalence of ideal cardiovascular health by educational attainment and length of stay in the U.S. in the three subgroups of Asians. Secondly, we fit binary logistic regression models to examine the association between education and cardiovascular health. We fit a crude model with education as the main predictor, followed by models that adjusted for age, sex at birth, income, and nativity status. Thirdly, we tested for effect measure modification by the length of stay in the U.S. (less than 10 years, 10 years or more) on the association between education and CVH. SAS 9.4 (SAS Institute Inc., Cary, NC, USA) was used for all statistical analyses. All statistical testing was two-sided at the 5% significance level and accounted for sampling probability.

## 3. Results

Table 1 shows the demographic distribution of the Asian American sample by nativity and length of stay in the U.S. (*n =* 1634). Foreign-born Asian Americans who lived in the U.S. <10 years were significantly younger than the U.S.-born (35.8 vs. 49.8, *p*-value < 0.0001) and were relatively more educated than their U.S.-born counterparts and those living in the U.S. 10 years or more (61.2% vs. 56.6% and 54.2% with college graduation or more, *p*-value < 0.001). A slightly higher percentage of U.S.-born Asians earned incomes of $75,000 or higher than foreign-born Asian Americans (53.2% vs. 49.9% and 41.0%, *p*-value < 0.0001). The U.S.-born were more likely to have completed some college than the foreign-born.

Diabetes prevalence among foreign-born Asian Americans living in the U.S. 10 or more years was 45.9% compared to 19.8% in foreign-born Asian Americans with a shorter stay in the U.S., and 20% among U.S.-born Asians (*p*-value < 0.0001). Applying sample-specific cutoffs for obesity, foreign-born Asian Americans staying in the U.S. less than 10 years had a slightly lower prevalence of obesity than foreign-born Asian Americans staying in the U.S. 10 or more years and U.S.-born Asians (37.5% vs. 41.9% and 45.5%, *p*-value < 0.0001). Foreign-born Asian Americans were less likely (*p*-value < 0.0001) to smoke (9.9% and 10.4% for ≥10 and <10 years in the U.S., respectively) than U.S.-born Asians (16%). Foreign-born Asian Americans staying in the U.S. ≥10 years had elevated or high blood pressure and elevated total cholesterol than Foreign-born Asian Americans staying <10 years in the U.S. and U.S.-born Asians (56.8% vs. 31.6% and 46.0%, *p*-value < 0.0001; 57.7% vs. 37.4% and 38.0%, *p*-value < 0.0001). U.S.-born Asians engaged in significantly (*p*-value < 0.05) more ideal physical activity (55.1%) than foreign-born Asian Americans (39.9% and 41.4% for ≥10 and <10 years in the U.S., respectively). For global CVH scores, foreign-born Asian Americans staying in the U.S. 10 years or more had significantly (*p*-value < 0.0001) decreased ideal CVH (≥6 metrics) prevalence (7.1%) than foreign-born Asian Americans staying less than 10 years and U.S.-born Asians (17.1% and 15.9% for foreign-born Asian Americans staying less than 10 years and U.S.-born Asians, respectively).

**Figure 1:**
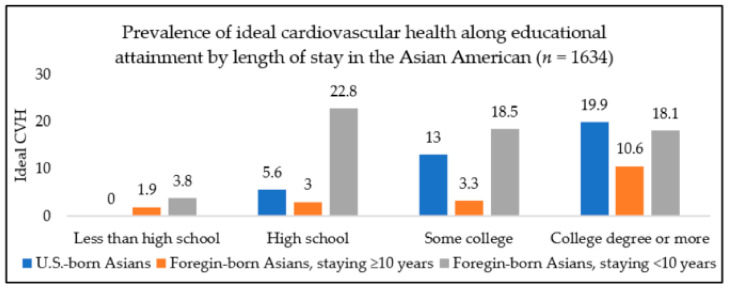
Prevalence of ideal cardiovascular health by education and length of stay among Asian Americans, NHANES (National Health and Nutrition Examination Survey) 2011–2016. CVH, cardiovascular health. Note: No U.S.-born Asian American participant with less than a high school education had ideal cardiovascular health.

Figure 1 displays the prevalence of ideal cardiovascular health by educational attainment and length of stay in the U.S. The prevalence increased with higher education (*p* for trend <0.05), reaching 18.1% among those with a college degree or more education compared to 3.8% among less than high school educated foreign-born Asian Americans staying in the U.S. less than 10 years. The prevalence of having ideal CVH also increased with higher education (*p* for trend <0.05) in foreign-born Asian Americans staying in the U.S. 10 years or more but to a lower degree (reaching 10.6% among those with a college degree or more education compared to 1.9% among less than high school educated). A similar pattern was observed for U.S.-born Asian Americans (*p* for trend <0.05), although all U.S.-born participants had completed at least a high school education or higher.

Results from the multivariable logistic regression analysis are shown in Table 2. Results indicate that in the unadjusted model (model 1), the odds of ideal cardiovascular health for Asian Americans was 85% lower for those with less than a high school education compared to those with a college degree (odds ratio (OR): 0.15, 95% confidence interval (CI): 0.05–0.46, *p*-value < 0.001). This pattern of lower odds of ideal CVH remained in all models, except for the less than high school group in the final model (model 3) that adjusted for income and nativity. For example, in model 2 adjusting for age and sex at birth, odds of ideal CVH was 73% lower in those with the lowest vs. highest levels of education (OR = 0.27, 95% CI: 0.08–0.85, *p*-value < 0.05). In model 3, the odds of ideal CVH were 66% lower in the less than high school education group compared to the college-educated group but were no longer significant (odds ratio (OR): 0.34, 95% confidence interval (CI): 0.10–1.13, *p*-value > 0.05).

Table 3 shows stratified analyses of the association between educational attainment and ideal CVH by the length of stay in the U.S. for the foreign-born only (*p*-value for interaction >0.05). Among foreign-born Asian Americans who lived in the U.S. less than 10 years, those with less than a high school education had an 82% lower odds of ideal CVH compared to their college-educated counterparts (Model 1: OR = 0.18, 95% CI: 0.04–0.85, *p*-value < 0.05). Asian Americans who lived 10 years or more in the U.S. showed a similar pattern of worse health for the least vs. most highly educated immigrants. Specifically, the odds of ideal CVH among those with less than high school education was 84% lower when compared to college or more educated participants (Model 1: OR= 0.16, 95% CI: 0.03–0.77, *p*-value < 0.05). The magnitude and direction of this association remained in fully adjusted models for both short and long-term immigrants, although results became non-significant (*p*-value > 0.05).

## 4. Discussion

Asian American immigrants constitute a large and growing segment of the U.S. population, and preventive strategies are needed to reduce cardiovascular disparities in this population. Our study addressed gaps in the literature on the role of social factors in shaping cardiovascular health in Asian Americans. Using data from a large population-based sample, we found that less than twenty-percent of Asian American adults had an ideal CVH score. Moreover, we found a significantly increased prevalence of ideal CVH with an increasing level of educational attainment in both U.S. and foreign-born Asian Americans. In models stratified by length of stay in the United States, this educational gradient remained, although results were more pronounced for those living in the U.S. ≥10 years and became non-significant in fully adjusted models.

Our study supported earlier research examining CVH among Asian Americans [7,16,51]. However, these studies either indirectly examined the role of education (i.e., adjusted for education), examined only select CVH metrics, or did not examine the role of education on CVH among Asian Americans specifically. In our study, we examined if education showed gradients in the composite cardiovascular health score and thus contributed to a handful of studies assessing the association between education and cardiovascular health, as defined by the American Heart Association [7,13,16]. Over the past few decades, research on the health of Asian Americans has suffered from stereotypes on the ‘minority myth’ model. The minority myth model is based on stereotypes of Asian Americans as self-sufficient, all being well-educated, and with a lower burden of disease [52]. The myth was mainly perpetuated after the immigration act of 1965 when many Asian American immigrants arriving in the U.S. were highly skilled and educated [53]. However, similar to other research studies, we found health disparities within Asian Americans and by subgroups, such as the U.S. vs. foreign-born [7,54,55]. Other researchers have similarly urged the importance of disaggregating data among Asian subgroups in order to disprove the minority myth stereotype and advance understanding of the complexity of Asian American health [6,53]. Moreover, as a growing population in the U.S., there is a need to demonstrate the pervasiveness of education and income on population health. As Contreras et al. showed [56], while national overall health has progressed in the U.S., individuals with little education and low annual income are less likely to reap the benefits of good health. 

Differences in the association between education and cardiovascular health by the length of stay in the U.S. were not consistent in our study population. Although the odds of ideal CVH showed an educational gradient for both foreign and U.S.-born Asian Americans, the odds of having ideal CVH was substantially lower for those in low vs. high education among the foreign-born living in the U.S. more than 10 years. However, for both groups, results became non-significant after adjusting for confounders. A number of prior studies have shown that foreign-born Asian Americans who reported living in the United States for more than 10 years were more likely to have CVH problems (e.g., hypertension, diabetes, obesity) than recent immigrants [7,57]. However, some studies have found no significant difference by the length of stay after adjusting for demographic and health characteristics or have shown that cardiovascular risk factors are lower among those living in the United States >10 years [6,58,59]. These findings demonstrate the heterogeneity and complexity of this relationship and demand data and research on specific aspects of acculturation proxies (the language spoken at home, age since migration, cultural belief, and access to care) in future research.

## 5. Limitation

Our study has some limitations that warrant attention. As a cross-sectional study, we did not examine if CVH deteriorates over time due to longer stay in the United States, limiting causal inference of our associations. Further, due to data limitations, we were unable to disaggregate cardiovascular health by the various ethnic groups that comprise Asian Americans. A growing number of studies report varying CVD risk across Asian groups, likely due to the distinct cultural, social, and integration patterns of Asian Americans in the U.S. society [60,61]. Our study may also have been underpowered to detect differences in educational gradients in CVH heath by the length of stay in the United States. Moreover, length of stay in the U.S. is a proxy measure of ‘acculturation’-related processes that shape health in immigrant groups. Further research is needed using more diverse and contextual measures, such as language spoken at home, age at migration, cultural beliefs, and immigration policies and racial/ethnic discrimination experiences, including occupational segregation, which can better elucidate the intersection of migration and contextual effects on health [6,16]. A final limitation is that education is only one component of the socioeconomic position that may influence cardiovascular health. Other comprehensive measures (e.g., accumulated wealth) were not available in our study data.

## 6. Conclusions

Our study explicitly examined educational attainment patterns in cardiovascular health among Asian Americans. We found that compared to highly educated Asian Americans, the odds of ideal CVH in poorly educated Asians were substantially lower. These results remained statistically significant after adjusting for confounders except when controlling for nativity status. Our results also showed that models stratified by time in the U.S. were less consistent but showed similar education gradients in CVH. Another important strength of our study was the use of a nationally representative survey with a large number of Asian Americans that allowed us to include both self-reported and laboratory-confirmed cardiovascular health data. We showed that compared to other population groups, low educational attainment was a risk factor for cardiovascular health. These findings refute notions of a lack of social gradient in CVH for Asian American populations. Results also showed the heterogeneity of Asian American populations and the need to consider education and length of stay in the U.S. as important factors for the prevention of cardiovascular disease and meeting goals to reduce inequities in cardiovascular health. 

## Figures and Tables

**Table 1:** Weighted demographic characteristics of Asian Americans by nativity and length of stay, NHANES 2011–2016 (n = 1634).

Characteristics	Asians, U.S.-Born (*n =* 226)	Asians, Foreign-Born, ≥10 Years (*n =* 993)	Asians, Foreign-Born, <10 Years (*n =* 404)	*p*-Value *
		% (Weighted Frequency), *n*		
Age mean (SE)	36.2 (1.46)	49.8 (0.78)	35.8 (1.00)	<0.0001
Age category (years)				<0.0001
20–39	69.7 (152)	25.6 (236)	72.3 (277)	
40–64	20.1 (48)	56.3 (572)	22.5 (102)	
65+	10.4 (26)	18.2 (185)	5.2 (25)	
Sex at birth				0.123
Male	50.7 (121)	45.9 (481)	51.3 (216)	
Female	49.3 (105)	54.1 (512)	48.7 (188)	
Educational level				<0.0001
Less than high school	1.7 (5)	13.2 (141)	13.0 (57)	
High school	10.2 (22)	13.1 (137)	11.7 (51)	
Some college education	31.5 (70)	19.5 (194)	14.1 (61)	
College graduate or more	56.6 (129)	54.2 (521)	61.2 (235)	
Income level				<0.0001
$0–$24,999	10.1 (24)	13.3 (134)	14.6 (57)	
$25,000–$74,999	37.7 (78)	37.8 (353)	44.4 (172)	
$75,000+	53.2 (113)	49.9 (443)	41.0 (145)	
HbA1c (%)				<0.0001
HbA1c less than 6.5%	80.0 (174)	54.1 (499)	80.3 (298)	
HbA1c more than 6.5%	20.0 (46)	45.9 (450)	19.8 (88)	
BMI (kg/m^2^) (Asian cut off)				<0.0001
Non obese (18.5 to <25)	54.5 (126)	58.1 (566)	62.5 (253)	
Obese (≥25)	45.5 (99)	41.9 (418)	37.5 (150)	
Diet score				<0.0001
Ideal (≥81)	4.3 (10)	4.6 (45)	3.2 (13)	
Poor (0–80)	95.7 (215)	95.4 (932)	96.8 (388)	
Blood pressure (mm of Hg)				<0.0001
Ideal (systolic <120, diastolic <80)	54.0 (120)	43.2 (405)	68.4 (257)	
Poor or elevated (systolic >120, diastolic >80)	46.0 (104)	56.8 (554)	31.6 (133)	
Smoking				<0.0001
Ideal (never smoked or quit >1 year)	84.0 (191)	90.1 (890)	89.6 (360)	
Poor (current smoker or quit <1 year)	16.0 (35)	9.9 (103)	10.4 (44)	
Total Cholesterol (mg/dl)				<0.0001
Ideal (<200)	62.0 (134)	42.3 (377)	62.7 (235)	
Poor (>200)	38.0 (85)	57.7 (556)	37.4 (147)	
Physical activity (PA)				<0.05
Ideal (≥75 min vig. or ≥150 min mod PA/wk)	55.1 (125)	39.9 (390)	41.4 (161)	
Poor (<75 min vig. or <150 min mod PA/wk)	44.9 (101)	60.2 (603)	58.6 (243)	
CVH metrics **				<0.0001
Ideal (6 or more metrics)	15.9 (35)	7.1 (54)	17.1 (56)	
Non-ideal (0–5 metrics)	84.1 (180)	92.9 (828)	82.9 (310)	

SE, standard error; BMI, body mass index; HbA1c, glycated hemoglobin; PA, physical activity; CVH, cardiovascular health; NHANES, National Health and Nutrition Examination Survey; * *p*-value significant at <0.05; ** Ideal CVH based on the score of 6 or more on all CVH metrics and non-ideal CVH based on the score of 0–5.

**Table 2:** Association between educational level and ideal CVH ^a^ in Asian Americans, NHANES, 2011–2016 (n = 1634).

Educational Level	*N*	Model 1 (Crude) OR (95% CI) ^b^	Model 2 OR (95% CI) ^b^	Model 3 OR (95% CI) ^b^
Less than high school	203	0.15 (0.05, 0.46)	0.27 (0.08, 0.85)	0.34 (0.10, 1.13)
High school	210	0.55 (0.31, 0.98)	0.64 (0.36, 1.16)	0.78 (0.41, 1.48)
Some college	325	0.55 (0.35, 0.86)	0.52 (0.34, 0.79)	0.52 (0.34, 0.80)
College degree or more	885	1.00	1.00	1.00

Note: ^a^ Ideal CVH, ideal cardiovascular health: outcome (score of 6 or more on CVH metrics); ^b^ OR = odds ratio, CI = confidence interval; Model 1—crude; Model 2—adjusted for age and sex at birth; Model 3—adjusted for model 2 plus income category and nativity status.

**Table 3:** Association between education and ideal CVH ^a^ by the length of stay in the U.S. among Asian Americans, NHANES, 2011–2016 (n = 1397).

Educational Level	Length of Stay in U.S. Less than 10 Years (*n =* 404)				Length of Stay in U.S. 10 Years or More (*n =* 993)			
	*n*	Model 1 (Crude) OR (95% CI) ^b^	Model 2 OR, (95% CI) ^b^	Model 3 OR, (95% CI) ^b^	*n*	Model 1 (Crude) OR (95% CI) ^b^	Model 2 OR, (95% CI) ^b^	Model 3 OR, (95% CI) ^b^
Less than high school	57	0.18 (0.04, 0.85)	0.34 (0.06, 1.92)	0.45 (0.09, 2.35)	141	0.16 (0.03, 0.77)	0.24 (0.05, 1.18)	0.36 (0.06, 1.94)
High school	51	1.33 (0.53, 3.36)	1.50 (0.57, 3.94)	2.09 (0.72, 6.04)	137	0.26 (0.08, 0.88)	0.30 (0.09, 1.04)	0.41 (0.12, 1.43)
Some college	61	1.02 (0.52, 2.02)	1.08 (0.55, 2.12)	1.14 (0.55, 2.39)	194	0.28 (0.11, 0.77)	0.23 (0.10, 0.55)	0.23 (0.11, 0.51)
College degree or more	235	1.00	1.00	1.00	521	1.00	1.00	1.00

Note: ^a^ Ideal CVH, ideal cardiovascular health: outcome (6 or more metrics); ^b^ OR = odds ratio, CI = confidence interval; Model 1—crude; Model 2—adjusted for age and sex at birth; Model 3—adjusted for model 2 plus income category.

## Data Availability

Publicly available datasets were analyzed in this study. All Data and materials are publicly available at the National Center for Health Statistics (NCHS) website (https://cdc.gov/nchs/nhanes/about_nhanes.htm).
